# Supplementary material for: Growth Performance, Metabolomics, and Microbiome Responses of Weaned Pigs Fed Diets Containing Growth-Promoting Antibiotics and Various Feed Additives
Source: Animals (Basel). 2023 Dec 23;14(1):60. doi: 10.3390/ani14010060 (PMC10778031; doi:10.3390/ani14010060)
Supplement: Supplementary file 1 [file animals-14-00060-s001.zip › animals-2704068-supplementary.pdf]

Table S1. Summary of chemical compounds identified in chemometrics analysis of feed additives.

**Chemical compounds***	**Essential oil and herbal blends**			**Phytogenic extracts**				**Milk-derived substances**		**Yeast products**		
	**PHY01**	**PHY02**	**PHY03**	**TUM**	**GAR**	**BOE**	**SOE**	**VM01**	**VSM02**	**YN01**	**YN02**	**YC03**
Free amino acids										+	+	+
Choline	+	+	+									
Glycine	+	+	+									
Pipecolic acid	+	+	+									
Dipeptides										+	+	+
Betaine	+	+	+			+						
Flavonoids (nobiletin, tangeritin, quercetin)						+	+					
Piperine	+											
Quinic acid									+			
Curcumin		+		+								
Ibervirin					+							

* Feed additives were proprietary products of essential oil and herbal blends (PHY01, PHY02, PHY03); phytogenic extracts comprised of turmeric (TUM), garlic (GAR), bitter orange (BOE), and sweet orange (SOE); volatile milk-derived substances (VM01) and volatile and semi-volatile milk-derived substances (VSM02); yeast nucleotide mixtures (YN01, YN02) and a yeast cell wall mixture (YC03). Additives designated with + contained one or more of the chemical compounds listed.

Table S2. Serum amino acid concentrations of pigs fed antibiotics, essential oils and herbal blends, and phytogenic extracts from experiment 1.

Amino acid, µM	Dietary treatments[1]							*P*-value[2]
	NC	PC	PHY01	PHY02	PHY03	TUM	GAR	
Alanine	351	363	414	339	363	320	391	0.466
Arginine	263	305	306	237	277	320	391	0.734
Asparagine	116	128	163	125	122	95	149	0.610
Aspartic acid	4	4	5	4	5	6	5	0.707
Citrulline	20	35	36	32	29	27	29	0.504
Glutamic acid	172	189	193	185	212	210	238	0.845
Glutamine	728	798	824	728	792	664	820	0.909
Glycine	1,411	1,515	1,492	1,277	1,508	1,252	1,414	0.956
Histidine	43	57	55	42	50	39	45	0.677
Leucine-Isoleucine	239	335	358	245	311	282	309	0.883
Lysine	285	335	358	245	311	282	309	0.883
Methionine	22	20	22	20	17	17	18	0.880
Ornithine	74	91	91	77	85	71	86	0.835
Phenylalanine	125	147	144	128	126	107	122	0.533
Proline	484	507	539	455	517	425	523	0.778
Serine	175	185	226	204	181	153	220	0.646
Taurine	22	51	32	22	41	33	24	0.267
Threonine	429	351	351	293	262	251	339	0.132
Tryptophan	53	65	64	46	52	45	48	0.539
Tyrosine	82	95	94	75	74	60	82	0.365
Valine	280	321	316	246	274	240	283	0.409

[1] Dietary treatments include antibiotic (PC), no antibiotic (NC), essential oil and herbal blend 1 (PHY01), essential oil and herbal blend 2 (PHY02), essential oil and herb blend 3 (PHY03), turmeric (TUM), and garlic (GAR).

[2] *P*-value from Kruskal Wallis one-way analysis of variance.

Table S3. Serum amino acid concentrations of pigs fed antibiotics, phytogenic extracts, and milk-derived substances from experiment 2.

Amino acid, µM	Dietary treatments[1]						*P*-value[2]
	NC	PC	BOE	SOE	VM01	VSM02	
Alanine	504	529	482	542	438	468	0.385
Arginine	292	345	297	337	283	285	0.340
Asparagine	123	148	139	141	107	117	0.818
Aspartic acid	5	6	6	7	5	7	0.607
Citrulline	31	32	32	38	38	35	0.884
Glutamic acid	247	181	234	246	176	235	0.406
Glutamine	749	892	813	973	768	779	0.078
Glycine	1,594	1,745	1,756	1,621	1,461	1,682	0.258
Histidine	56	77	54	65	57	55	0.369
Leucine-Isoleucine	299	325	282	313	272	294	0.771
Lysine	387	444	335	404	292	367	0.301
Methionine	15	15	13	22	9	12	0.338
Ornithine	120	128	116	128	102	126	0.764
Phenylalanine	142	178	143	170	156	138	0.556
Proline	597	635	566	626	524	547	0.203
Serine	254	317	241	320	211	259	0.362
Taurine	124	223	117	148	123	161	0.127
Threonine	385	451	402	484	349	333	0.224
Tryptophan	78	87	67	79	69	60	0.551
Tyrosine	81	91	72	94	76	71	0.562
Valine	364	421	342	365	315	356	0.432

[1] Dietary treatments include antibiotic (PC), no antibiotic (NC), bitter orange extract (BOE), sweet orange extract (SOE), volatile milk-derived substances (VM01), and volatile and semi-volatile milk-derived substances (VSM02).

[2] *P*-value from Kruskal Wallis one-way analysis of variance.

Table S4. Serum amino acid concentrations of pigs fed antibiotics and yeast products from experiment 3.

Amino acid, μM	Dietary treatments[1]					*P*-value[2]
	NC	PC	YN01	YN02	YC03	
Alanine	430	391	400	444	414	0.444
Arginine	266	298	264	255	252	0.144
Asparagine	140	136	126	142	134	0.993
Aspartic acid	7	6	5	5	6	0.427
Citrulline	47	41	57	46	31	0.118
Glutamic acid	291	316	236	259	245	0.649
Glutamine	749	764	689	713	683	0.517
Glycine	1,508	1,605	1,481	1,642	1,502	0.899
Histidine	49	66	55	55	54	0.580
Leucine-Isoleucine	263	285	268	285	261	0.735
Lysine	285[a]	424[b]	330[a]	329[a]	300[a]	0.037
Methionine	18	16	23	15	17	0.860
Ornithine	120	129	123	123	115	0.849
Phenylalanine	126	154	120	120	132	0.207
Proline	505	510	479	511	489	0.868
Serine	190	206	201	239	246	0.120
Taurine	27	34	50	54	19	0.210
Threonine	241	284	280	258	249	0.924
Tryptophan	58	72	61	58	59	0.548
Tyrosine	64	58	64	65	65	0.972
Valine	272	327	315	293	283	0.351

[1] Dietary treatments include antibiotic (PC), no antibiotic (NC), yeast nucleotide product 1 (YN01), yeast nucleotide product 2 (YN02), and yeast cell wall (YC03) product.
[2] *P*-value from Kruskal Wallis one-way analysis of variance.
[a,b] Means within a row with uncommon superscripts differ ($P < 0.05$).

Table S5. Serum metabolites with high abundance and specificity to pigs fed antibiotics, essential oils and herbal blends, phytogenic extracts, milk-derived substances, and yeast products compared with pigs fed the negative control diets.

Metabolite[1]	Metabolite identification[2]	Experiment	Treatment[3]	Indicator value[4]	Negative control mean[5] ± standard deviation	Treatment mean[5] ± standard deviation	*P*-value[6]
3.31_321.1015 DC		1	PC	0.997	0 ± 0	0.007 ± 0.002	0.001
3.04_279.0921 HQ	Sulfadimidine	1	PC	0.996	0 ± 0.001	0.055 ± 0.021	0.001
3.04_279.0921 HQ	Sulfadimidine	2	PC	0.985	0.001 ± 0.003	0.066 ± 0.017	0.000
7.90_221.0204 HQ		2	BOE	0.875	0 ± 0.001	0.003 ± 0.002	0.002
3.04_279.0921 HQ	Sulfadimidine	3	PC	0.980	0.001 ± 0.003	0.062 ± 0.014	0.001
4.45_264.0803 DC		3	PC	0.875	0.003 ± 0	0.079 ± 0.059	0.003

[1]Metabolite characteristics listed as retention time-mass method of derivatization used [either negative mode (ne), positive mode (po), dansyl chloride (DC), or 2-hydrazinoquinoline (HQ)].

[2]Metabolite identification based on database search of the adjusted mass. Identification was left blank if no metabolite was identified with this mass.

[3]Dietary treatments include antibiotics (PC), bitter orange extract (BOE).

[4]The indicator value is a product of frequency and abundance and ranges from 0 to 1. Greater indicator values show that a given taxon is more abundant in all samples from a given group compared with other groups. The table shows indicator values greater than 0.8, indicating that the selected metabolites are faithful biomarkers of a given treatment.

[5]Means expressed as relative abundance with respect to all metabolites (%).

[6]*P*-value from Kruskal Wallis one-way analysis of variance.

Table S6. Effects of dietary antibiotics, essential oil and herbal extracts, and phytogenic extracts on amino acid, short chain fatty acid, and bile acid concentrations in cecal contents from experiment 1.

Analyte	Dietary treatments[1]							*P*-value[2]
	NC	PC	PHY01	PHY02	PHY03	TUM	GAR	
**Amino acids, mg/g**								
Alanine	0.597	0.640	0.491	0.609	0.740	0.726	0.497	0.551
Arginine	0.017	0.015	0.008	0.015	0.007	0.123	0.008	0.675
Asparagine	0.001	0.000	0.000	0.000	0.000	0.000	0.000	0.806
Aspartic acid	0.275	0.317	0.212	0.286	0.277	0.188	0.198	0.580
Citrulline	0.006	0.024	0.011	0.020	0.016	0.007	0.003*	0.181
Glutamic acid	0.966	1.182	0.971	0.782	1.104	0.886	1.410	0.941
Glutamine	0.003	0.004	0.002	0.003	0.002	0.087	0.001	0.921
Glycine	0.090	0.122	0.074	0.083	0.110	0.131	0.094	0.916
Histidine	0.006	0.008	0.003	0.004	0.005	0.030	0.004	0.830
Leucine-Isoleucine	0.044	0.028	0.022	0.043	0.038	0.074	0.017	0.312
Lysine	0.171	0.147	0.112	0.164	0.177	0.146	0.103	0.491
Methionine	0.003	0.002	0.001	0.002	0.002	0.003	0.001	0.081
Ornithine	0.002	0.002	0.005	0.005	0.014*	0.003	0.008	0.079
Phenylalanine	0.069	0.053	0.044	0.064	0.065	0.107	0.037	0.403
Proline	0.060	0.047	0.039	0.060	0.074	0.082	0.034*	0.229
r-amino-n-butyric	0.003	0.003	0.004	0.002	0.003	0.002	0.002	0.664
Serine	0.029	0.023	0.014	0.029	0.020	0.079	0.009*	0.275
Taurine	0.000	0.001	0.000	0.000	0.001	0.003	0.000	0.240
Threonine	0.040	0.040	0.027	0.040	0.034	0.068	0.019	0.641
Tryptophan	0.002	0.001	0.000	0.001	0.001	0.016	0.001	0.735
Tyrosine	0.099	0.057	0.039	0.082	0.075	0.150	0.036	0.328
**Short chains fatty acids, mg/g**								
Acetic acid	29.6	32.4	30.1	23.7	24.1	24.4	23.3	0.562
Propionic acid	20.8	21.0	17.4	14.3	16.3	15.8	14.4	0.624
Butyric acid	17.4	22.2	18.1	14.8	15.5	15.5	15.0	0.603
Valeric acid	3.67	6.05	4.00	3.50	4.26	3.56	3.36	0.573
Isovaleric acid	124.6	57.5	45.7	49.0	130.3	58.1	23.9	0.692
**Bile acids, μg/g**								
Cholic acid	0.05	0.10	0.15	0.00	0.05	0.00	0.00	0.515

Chenodeoxycholic acid	11.9	19.5	10.0	26.3	36.6	72.3	21.5	0.846
Lithocholic acid	101.2	111.3	95.1	119.2	99.1	130.1	55.1	0.379
Taurochenodeoxycholic acid	0.19	0.13	0.13	0.00*	1.81	0.63	0.06	0.091
Hyodeoxycholic acid	413.8	495.3	485.9	576.4	454.3	564.0	405.7	0.801

[1] Dietary treatments include antibiotic (PC), no antibiotic (NC), essential oil and herbal blend 1 (PHY01), essential oil and herbal blend 2 (PHY02), essential oil and herb blend 3 (PHY03), turmeric (TUM), and garlic (GAR).

[2] P-value from Kruskal Wallis one-way analysis of variance.

* Significantly different from negative control ($P < 0.1$).

Table S7. Effects of dietary antibiotics, phytogenic extracts, and milk-derived substances on amino acid, short chain fatty acid, and bile acid concentrations in cecal contents from experiment 2.

Analyte	Dietary treatments[1]						
	NC	PC	BOE	SOE	VM01	VSM02	*P*-value[2]
**Amino acids, mg/g**							
Alanine	0.301	0.283	0.406	0.349	0.406	0.279	0.474
Arginine	0.006	0.006	0.035	0.003	0.005	0.003	0.581
Asparagine	0.000	0.000	0.044	0.000	0.000	0.000	0.445
Aspartic acid	0.226	0.171	0.271	0.206	0.175	0.220	0.474
Citrulline	0.006	0.001	0.003	0.004	0.001	0.007	0.284
Glutamic acid	0.679	0.558	0.712	0.722	0.572	0.805	0.474
Glutamine	0.001	0.001	0.047	0.004	0.001	0.001	0.170
Glycine	0.035	0.030	0.085	0.050	0.034	0.049	0.474
Histidine	0.002	0.002	0.019	0.005	0.003	0.002	0.268
Leucine-Isoleucine	0.013	0.008	0.038	0.011	0.011	0.012	0.670
Lysine	0.081	0.063	0.145	0.063	0.059	0.087	0.474
Methionine	0.001	0.000	0.005	0.001	0.001	0.001	0.805
Ornithine	0.007	0.001	0.033	0.003	0.005	0.009	0.241
Phenylalanine	0.025	0.023	0.053	0.023	0.026	0.026	0.495
Proline	0.026	0.026	0.053	0.032	0.027	0.026	0.522
r-amino-n-butyric	0.004	0.001	0.003	0.004	0.004	0.003	0.299
Serine	0.010	0.005	0.044	0.007	0.006	0.007	0.454
Taurine	0.000	0.004	0.003	0.001	0.006	0.002	0.203
Threonine	0.027	0.017	0.039	0.022	0.018	0.019	0.509
Tryptophan	0.001	0.001	0.007	0.000	0.001	0.001	0.213
Tyrosine	0.048	0.032	0.102	0.034	0.052	0.042	0.435
**Short chains fatty acids, mg/g**							
Acetic acid	33.4	34.2	29.9	28.6	26.4*	32.7	0.548
Propionic acid	20.0	21.9	18.7	16.8	16.2*	22.3	0.372
Butyric acid	22.2	25.0	19.2	17.8*	17.2*	20.2	0.131
Valeric acid	5.3[ab]	7.5[a]	4.5[ab]	4.4[ab]	4.2[b]	4.5[ab]	0.039
Isovaleric acid	22.5	66.7	48.2	129.9	60.1	157.9	0.164

**Bile acids, μg/g**							
Cholic acid	0.00	0.00	0.20	0.00	0.00	0.00	0.394
Chenodeoxycholic acid	17.8	8.0	42.6	21.3	17.1	65.9	0.538
Lithocholic acid	59.0	138.3*	76.1	96.6	71.0	110.6	0.279
Taurochenodeoxycholic acid	0.40	0.90	0.10	0.10	0.50	7.3	0.385
Hyodeoxycholic acid	393.4	472.1	455.8	500.1	366.6	457.2	0.898

[1] Dietary treatments include antibiotic (PC), no antibiotic (NC), bitter orange extract (BOE), sweet orange extract (SOE), volatile milk-derived substances (VM01), and volatile & semi-volatile milk-derived substances (VSM02).

[2] P-value from Kruskal Wallis one-way analysis of variance.

* Significantly different from negative control ($P < 0.1$).

[a,b] Means within a row with uncommon superscripts differ ($P < 0.05$).

Table S8. Effects of dietary antibiotics and yeast products on amino acid, short chain fatty acid, and bile acid concentrations in cecal contents from experiment 3.

Analyte	Dietary treatments[1]					*P*-value[2]
	NC	PC	YN01	YN02	YC03	
**Amino acids, mg/g**						
Alanine	0.493	0.634	0.382	0.405	0.502	0.060
Arginine	0.002	0.002	0.001	0.001	0.005	0.095
Asparagine	0.000	0.000	0.000	0.000	0.000	0.694
Aspartic acid	0.203	0.266	0.129	0.205	0.204	0.196
Citrulline	0.006	0.013	0.002	0.004	0.001*	0.051
Glutamic acid	0.642[b]	0.982[a]	0.360[c]	0.582[b]	0.586[b]	0.046
Glutamine	0.000	0.000	0.000	0.000	0.001	0.179
Glycine	0.045	0.068	0.027	0.033	0.050	0.175
Histidine	0.001[b]	0.002[a]	0.001[b]	0.001[b]	0.001[b]	0.039
Leucine-Isoleucine	0.020[b]	0.030[a]	0.013[b]	0.018[b]	0.022[b]	0.032
Lysine	0.095[b]	0.128[a]	0.047[b]	0.083[b]	0.087[b]	0.013
Methionine	0.001	0.002	0.001	0.001	0.002	0.076
Ornithine	0.002	0.006	0.001	0.001	0.003	0.396
Phenylalanine	0.036[b]	0.046[a]	0.023[b]	0.030[b]	0.039[b]	0.012
Proline	0.039	0.042	0.029	0.037	0.038	0.351
r-amino-n-butyric	0.014[a]	0.015[a]	0.005[b]	0.012[a]	0.015[a]	0.028
Serine	0.000	0.001	0.001	0.000	0.000	0.407
Taurine	0.019	0.025	0.010	0.021	0.022	0.103
Threonine	0.000	0.001	0.000	0.000	0.000	0.059
Tryptophan	0.023	0.034	0.012	0.017	0.025	0.126
Tyrosine	0.027[b]	0.045[a]	0.015[b]	0.023[b]	0.029[b]	0.004
**Short chains fatty acids, mg/g**						
Acetic acid	36.1	41.2	37.0	36.8	42.0	0.480
Propionic acid	23.7	26.9	24.0	23.8	25.3	0.574
Butyric acid	27.3	28.5	26.1	25.0	30.9	0.340
Valeric acid	7.4	7.7	7.1	6.4	8.9	0.181
Isovaleric acid	19.2	32.8	9.3	6.1	9.0	0.979

**Bile acids, μg/g**						
Cholic acid	0.00[b]	0.36[a]	0.00[b]	0.00[b]	0.00[b]	0.002
Chenodeoxycholic acid	15.5	30.0	15.2	8.7	7.6	0.775
Lithocholic acid	151.3	151.9	126.5	115.7	117.0	0.535
Taurochenodeoxycholic acid	0.00	0.00	0.06	0.00	0.00	0.406
Hyodeoxycholic acid	697.8	764.6	614.3	542.5	589.8	0.981

[1] Dietary treatments include antibiotic (PC), no antibiotic (NC), yeast nucleotide product 1 (YN01), yeast nucleotide product 2 (YN02), and yeast cell wall (YC03) product.

[2] P-value from Kruskal Wallis one-way analysis of variance.

* Significantly different from negative control ($P < 0.1$).

[a,b,c] Means within a row with uncommon superscripts differ ($P < 0.05$).

Table S9. Cecal metabolites with high abundance and specificity to pigs fed antibiotics, essential oils and herbal blends, phytogenic extracts, milk-derived substances, and yeast products compared with pigs fed the negative control diets.

Metabolite[1]	Metabolite identification[2]	Experiment	Treatment[3]	Indicator value[4]	Negative control mean[5] ± standard deviation	Treatment mean[5] ± standard deviation	*P*-value[6]
2.95_279.0918_po	Sulfadimidine	1	PC	1.000	0 ± 0	0.764 ± 0.192	0.001
3.29_321.1024_po	N4-Acetylsulfadimidine	1	PC	1.000	0 ± 0	0.082 ± 0.051	0.001
3.74_479.1220_po	Chlortetracycline	1	PC	1.000	0 ± 0	0.275 ± 0.063	0.001
3.31_479.1221_po	Chlortetracycline	1	PC	1.000	0 ± 0	0.214 ± 0.076	0.001
4.28_479.1227_po	Chlortetracycline	1	PC	1.000	0 ± 0	0.356 ± 0.078	0.001
2.06_237.1603_po	Metabutethamine	1	PHY02	0.940	0.004 ± 0.003	0.064 ± 0.071	0.041
5.43_321.1267_dc	Butyramide	1	PHY03	0.834	0.033 ± 0.038	0.168 ± 0.071	0.001
6.31_300.1031_dc		1	PHY03	0.815	0.034 ± 0.041	0.149 ± 0.067	0.002
1.76_181.9900_ne	Ibervirin	1	GAR	1.000	0 ± 0	0.051 ± 0.017	0.001
2.95_279.0918_po	Sulfadimidine	2	PC	1.000	0 ± 0	0.855 ± 0.169	0.001
3.29_321.1024_po	N4-Acetylsulfadimidine	2	PC	1.000	0 ± 0	0.094 ± 0.040	0.001
3.29_321.1024_po	N4-Acetylsulfadimidine	3	PC	1.000	0 ± 0	0.059 ± 0.026	0.001
3.74_479.1220_po	Chlortetracycline	3	PC	1.000	0 ± 0	0.197 ± 0.066	0.001
3.31_479.1221_po	Chlortetracycline	3	PC	1.000	0 ± 0	0.103 ± 0.029	0.001
4.28_479.1227_po	Chlortetracycline	3	PC	1.000	0.003 ± 0	0.286 ± 0.107	0.001
2.95_279.0918_po	Sulfadimidine	3	PC	0.993	0.003 ± 0.014	0.716 ± 0.170	0.001
4.09_331.2475_ne	Phloionolic acid	3	YN02	0.875	0.011 ± 0	0.048 ± 0.026	0.001

[1]Metabolite characteristics listed as retention time-mass method of derivatization used [either negative mode (ne), positive mode (po), dansyl chloride (DC), or 2-hydrazinoquinoline (HQ)].

[2]Metabolite identification based on database search of the adjusted mass. Identification was left blank if no metabolite was identified with this mass.

[3]Dietary treatments include antibiotics (PC), essential oil and herb mixture 2 (PHY02), essential oil and herb mixture 3 (PHY03), garlic (GAR), and yeast nucleotide 2 (YN02).

[4]The indicator value is a product of frequency and abundance and ranges from 0 to 1. Greater indicator values show that a given taxon is more abundant in all samples from a given group compared with other groups. The table shows indicator values greater than 0.8, indicating that the selected metabolites are faithful biomarkers of a given treatment.

[5]Means expressed as relative abundance with respect to all metabolites (%).

[6]*P*-value from Kruskal Wallis one-way analysis of variance.
